# Railway Axle Early Fatigue Crack Detection through Condition Monitoring Techniques

**DOI:** 10.3390/s23136143

**Published:** 2023-07-04

**Authors:** María Jesús Gomez, Cristina Castejon, Eduardo Corral, Marco Cocconcelli

**Affiliations:** 1 Mechanical Engineering Department, Avenida de la Universidad 30, 28982 Madrid, Spain; mjggarci@ing.uc3m.es (M.J.G.); ecorral@ing.uc3m.es (E.C.); 2 Department of Sciences and Methods for Engineering, University of Modena and Reggio Emilia, Via G. Amendola 2, 42124 Reggio Emilia, Italy; marco.cocconcelli@unimore.it

**Keywords:** railway axles, crack detection, automatic alarm value, change point analysis, clustering, Wavelet Packets Transform

## Abstract

The detection of cracks in rotating machinery is an unresolved issue today. In this work, a methodology for condition monitoring of railway axles is presented, based on crack detection by means of the automatic selection of patterns from the vibration signal measurement. The time waveforms were processed using the Wavelet Packet Transform, and appropriate alarm values for diagnosis were calculated automatically using non-supervised learning techniques based on Change Point Analysis algorithms. The validation was performed using vibration signals obtained during fatigue tests of two identical railway axle specimens, one of which cracked during the test while the other did not. During the test in which the axle cracked, the results show trend changes in the energy of the vibration signal associated with theoretical defect frequencies, which were particularly evident in the direction of vibration that was parallel to the track. These results are contrasted with those obtained during the test in which the fatigue limit was not exceeded, and the test therefore ended with the axle intact, verifying that the effects that were related to the crack did not appear in this case. With the results obtained, an adjusted alarm value for a condition monitoring process was established.

## 1. Introduction

It is well known that the appearance of a defect in a structural element generates a local flexibility, causing a change in its stiffness [1]. As a consequence, the natural frequencies are reduced and the modes of vibration are also altered, a fact that has traditionally been used to try to detect defects in structures. However, these techniques have a very important disadvantage, and that is that the variations in the natural frequency are negligible for practical needs. Another approach proposed in this regard is to use the ratio between acceleration and excitation amplitudes as a defect indicator, since defects cause changes in the transmissibility of forced vibrations, according to Akgun in [2]. These techniques also show limitations in practice when detecting defects in structures [3]. There are works that have focused on determining the transfer functions for some structures, as well as works from the amplitude and phase point of view, such as [4]. Deeper studies have allowed us to determine the impact of specific machinery on vibration levels in structures and connect specific events, for example, seismic noise [5].

In the case of rotating machinery, attempts have been made to use, as a means of diagnosis, changes in the dynamic vibration response when a defect appears. As a result, over the last six decades, numerous studies have been carried out on the dynamic behaviour of shafts and rotating machinery in the presence of a crack. The growing interest in this field is due to the fact that the breakage of such an element can cause a very critical situation, with high risks for people, the machine itself and surrounding equipment, but also an economic loss for the interruption of a service or a production.

The main field of application for these techniques is turbomachinery. According to works such as [6], the first subharmonic of the critical speed at which the crack shows up significantly is 1/4, which is an admissible speed in turbomachinery; however, there are many cases of rotating machinery in which it is not possible to reach such high speeds. Furthermore, although in theory these effects are very clear, in practice, early detection of a defect is not a simple task.

It has been widely documented in the literature that the frequency response of cracked rotors generates 1×, 2× and 3× harmonics of the rotational speed. The occurrence of these higher-order frequency harmonics has been explained as non-linear modulation effects due to the opening and closing of the crack; however, the intrinsic force driving the crack to generate harmonics is not entirely clear.

Recently, a new concept of Nonlinear Pseudo-Force (NPF) has been introduced that can explain the mechanism of generation of these harmonics from a new perspective [7].

It has also been pointed out that a cracked mechanical element experiences resonance in subharmonics and superharmonics. However, the phenomenon that causes this occurrence has not been fully explained, since the phenomenon of nonlinear vibration in an element with a breathing crack is not fully understood, and the inherent relationship between subharmonics and superharmonics with the crack parameters requires further investigation [8].

Recently, a new characteristic frequency found when a short steady-state disturbance occurs in a cracked rotor has been reported in the literature in [9]. In particular, the mixing effects of the rotor breathing and disturbance excitation periodically modulate the rotor stiffness. The study of the dynamic response to a disturbance introduces a new aspect to the study of the dynamic response of a cracked rotor.

As far as signal processing techniques are concerned, the difficulty in detecting certain theoretical effects of cracking in experimental signals has led to the replacement of the Fourier transform in favour of more advanced techniques. Above all, it has become common in recent years to use mathematical tools that provide information in both the time and frequency domains. Thus, numerous works have used the Hilbert–Huang transform [10,11], although the most widely used tool in recent times has undoubtedly been the Wavelet Transform (WT). In this paper [12], a review by the authors on the applications of the WT for rotating machinery diagnostics is presented.

The difficulties encountered in this field have also led to the use of artificial intelligence to perform classification tasks on the patterns obtained and thus make models for reliable diagnosis. In relation to the artificial intelligence tools that can be applied to classify patterns, the evolution had its beginnings in classical statistical decision approaches (deterministic and probabilistic) [13], from decision trees [14] to others deriving from the previous ones that were recently developed through computer packages. Also, fuzzy logic networks [15], or genetic algorithms, support vector machines and artificial neural networks, including hybrid versions of all of the above [16]. It is common to use these classifiers when it is known whether each piece of data corresponds to a sound or faulty mechanical element. In this way, classification systems are trained using a type of learning called supervised learning. Some works have combined WT with intelligent classification systems for diagnosis. Such is the case of works such as Sanz et al. [17], Bin et al. [18] or Gómez et al. in [19], where WT is combined with neural networks. In the case of Xiang et al. [20], the use of Genetic Algorithms (GAs) is included, and in the case of Hu et al. [21], an SVM-type classifier system is used.

However, there are cases where supervised learning is not possible, and only a history of machine lifetime data is available. For these cases where it is not known a priori to which group the data belong, there are also classification systems that follow unsupervised learning methods based on observable differences. In these cases, Change Point Analysis (CPA) is particularly useful. Change points are abrupt variations in time series. Such changes may represent transitions between states, such as the appearance of a defect. A review of these techniques can be found in [22].

Specifically, within the railway industry, scheduled predictive maintenance is mostly used at present, and in the case of axles, inspections are performed at intervals based on distance travelled. Condition monitoring-type solutions would be an invaluable aid to this industry in providing greater comfort and safety [23].

Research on condition monitoring of rail vehicles and track has gained a lot of importance in recent years. Typically, vibration signals taken from axle boxes are used. These types of signals are also used to measure degradation at track crossings [24], for track monitoring [25] or for condition monitoring of bogies in general [26]. Most work of this type has focused on the diagnosis of bearings, for which a review can be found in [27], and wheels, as in the case of [28].

However, railway axles are one of the most critical elements, and their failure can involve catastrophic consequences. Most research in this field is limited to modelling and simulation. Recent work in this area has studied the propagation of defects in railway axles, as in [29,30,31]. The work [32] analysed the performance of different vibration directions for the detection of cracks in railway axles using numerical simulations. It was concluded that the longitudinal direction (parallel to the track) was better than the vertical direction for this purpose. In the study [33], measurements of real railway axles were acquired on an axle installed in a fatigue-testing machine, concluding that the first three harmonics of the rotational speed could be used to detect axle defects using traditional signal processing methods such as Fast Fourier Transform.

Some advanced processing techniques, such as the Wavelet Transform, have been subsequently used for railway applications to assess the condition of track anchoring systems, such as in [24], or for bearing diagnosis, such as in [34]. In the case of axle diagnostics, WT was applied in [35,36] for the detection of cracks in railway axles. Specifically, in these papers, different intelligent classification systems were used to automate the diagnosis, such as neural networks in the case of [35] and support vector machines in the case of [36]. In these previous works using artificial intelligence, information related to the full unfiltered signal was used, and no study has been conducted on which specific patterns are optimal for crack diagnosis. Furthermore, in [36], a lack of generalisation of these algorithms was reported, since in this case there was a very large variation in the healthy behaviour between axles. Also, in this type of application, the conditions—such as load and speed—are not stationary. These things considered, in this field, it is rare to have experimental signals coming from a realistic, full-scale mechanical system with naturally generated fatigue cracks, which are more representative of reality.

In this work, a methodology is proposed to establish and validate a reliable condition monitoring technique for railway axles based on the analysis of vibration signals obtained during machine operation at sub-critical speeds. This includes a study and automatic selection of patterns based on a decision tree, which were then further analysed in depth for their relationship to theoretical fault frequencies. Then, a study of optimal patterns for early crack detection was performed among the selected, based on a clustering-based CPA study using the “kmeans ++” algorithm with unsupervised learning. The patterns that allowed an earlier detection were selected as optimal. Subsequently, an automatic adaptive alarm value was set for the selected optimal patterns as a threshold value to diagnose the crack. This allowed for generalisation, as it was based on the mean and standard deviation of the machine’s data, allowing it to adapt to different models and operating conditions. The validation of the methodology was performed on the basis of vibration signals obtained during fatigue tests of real full-scale railway axles. In addition, various vibration directions were measured. Therefore, the validation of the methodology may suggest the use of the proposed technique as a stopping criterion for fatigue tests of railway axles. The proposed methodology was based on signal processing using the Wavelet Packet Transform (WPT). The results are very promising for the establishment of a condition monitoring technique for railway axles. This approach can complement current inspection techniques, improving safety and reducing costs by extending inspection intervals.

The manuscript is sorted as follows: in the present section, an introduction focused on the problem to solve, state of the art and novelty of the work is presented; in Section 2, the change point analysis methods are presented in order to understand the adaptive threshold proposed. Next, in Section 3, the methodology to implement a condition monitoring system for early fatigue crack detection is presented, stablishing a set of hypotheses to be validated. In Section 4, the experimental setup used to validate the methodology is shown, and finally, the experimental results and conclusions are presented in Section 5 and Section 6.

## 2. Change Point Analysis (CPA) Methods

CPA algorithms are further classified into online and offline methods. Offline algorithms consider all the data at once and look backwards in time to try to identify the point of change. Online algorithms, by contrast, run with the process they are monitoring, processing each piece of data as it becomes available, with the added goal of detecting the change point as soon as possible after it occurs, ideally before the next piece of data arrives [37].

In practice, no CPA algorithm acts in real time because it needs to inspect new data before determining whether a change point has occurred. However, each online algorithm will need different amounts of data before claiming that change point detection has occurred.

Offline algorithms analyse the entire data set at once. However, real-time algorithms need to observe at least ε points forward from the change point candidate to assert that the change point has occurred. The value of ε depends on the nature of the algorithm. Online algorithms process data through a window of n points [38]. In this paper, an online CPA method is proposed based on observation of the whole data set.

### 2.1. Unsupervised Learning Methods

Unsupervised learning methods are typically used to discover patterns in unlabelled data. In the context of CPA analysis, these algorithms are used to segment time series based on the statistical characteristics of the data. The first documented methods use the likelihood ratio (LR) based on which of the probability density functions of two consecutive intervals are equal if they belong to the same state [38]. Probabilistic methods estimate the probability distribution of the new interval based on the observed data from the candidate change point [39]. In contrast, kernel-based (KB) methods bring the data into a higher dimensional space and detect change points by comparing the homogeneity of each sub-sequence [40]. Graph-based methods are recently introduced methods that represent the time series and apply statistical evaluations to detect change points [41].

Finally, clustering methods group the time series into their respective states and find the change points by identifying the transition from one state to another.

By means of clustering, a data series is divided into a set of subgroups *S* = {*S*_1_, *S*_2_, …, *S_k_*} classified according to their attributes, with each datum belonging exclusively to one of the subgroups, which form the total data together.

As a main classification for clustering types, there are hierarchical methods and partitioning methods, as proposed by Raftery and Fraley in [42]. Other classifications can be found in [43,44].

Partitioning clustering algorithms divide the data into subsets so that each datum belongs to only one subset. Although there are different and varied techniques [45], the most widely used is k-means clustering.

#### Cluster Analysis with “k-means ++” Algorithm

Cluster analysis, also called grouping, segmentation analysis or taxonomy analysis, partitions given sample data into groups or clusters. It is a type of unsupervised learning where clusters are formed in such a way that objects in the same cluster are similar and objects in different clusters are dissimilar. There are several clustering techniques and similarity measures to create the clusters.

The k-means algorithm or Lloyd’s algorithm [46] is one of the most popular clustering methods. Given a set of observations (*x*_1_, *x*_2_, …, *x_n_*), the algorithm can be formulated as an optimization problem whose objective is to construct *k* clusters (being *k* ≤ *n*) so that an objective function—for example, the total sum of the Euclidean distances $E\left(\mu_{i}\right)$ of the observations to their centroid *i* $\left(\mu_{i}\right)$ of each cluster *S* = {*S*_1_, *S*_2_, …, *S_k_*}—is minimized, according to Equation (1).

(1) $$E\left(\mu_{i}\right)=\overset{k}{\underset{i=1}{\sum }}\;\overset{n}{\underset{x_{j}\in S_{i}}{\sum }}\;\|x_{j}-\mu_{i}{\|}^{2}\;$$

The basic idea is that *k* centroids are chosen, and an initial non-optimal clustering is performed in *k* clusters (each cluster is composed of the data set that is closest to each of the selected centroids). The *k* centroids of each cluster are then recalculated, and each point is reallocated to its closest centroid. This loop is repeated until there are no new changes in the clusters, according to the following steps:

1. The *k* initial centres are chosen arbitrarily C = { , , …, }.
2. For each *i* ∈ {1, …, *k*}, set the cluster Si to be the set of observations that is closer to one than they are to the other for all *j* ≠ *i*.
3. For each *i* ∈ {1, …, *k*}, set it to be the centre of mass of all points in *Si*.
4. Steps 2 and 3 are repeated until C no longer changes.

The initialization task consists of choosing the first *k* clusters. Arthur and Vassilvtskii in [47] proposed an algorithm for the selection of *k* initial centroids based on probabilities. It is called k-means ++ and it is applied as follows:

1. Initizalization:
  a. An initial centre is chosen uniformly at random from X.
  b. The next centre is chosen via a weighting based on probabilities selecting = x′ ∈ X, with probability D(x) being the shortest distance from a data point x to the closest centre previously chosen.
  c. Step 1b is repeated until a total of *k* centres is chosen.
2–4. Same steps as for the standard k-means algorithm.

This algorithm improves the computational cost of Lloyd’s algorithm as well as the quality of the final solution, improving performance [48]. This algorithm has been used in the literature to detect the change point [49]. In the field of predictive maintenance applied to rotating machinery, this technique has also been used to detect the defect initiation point in bearings, as in the case of [50,51].

## 3. Hypothesis and Methodology

The state of the art, as well as the background on axle defectology in general and railway axles in particular, allows us to state that cracks can theoretically be detected in rotating elements. Therefore, the following hypotheses were established and were intended to be validated:

- Hypothesis 1: in vibratory signals obtained during axle rotation, there are certain frequency bands where an increase in energy is produced when a crack appears. The energy increases due to cracking are experimentally detectable through vibration signals obtained from the bearing housing using energy analysis by means of WPT.
- Hypothesis 2: these frequencies can be associated with the theoretical fault frequencies reviewed in the state of the art (harmonics of the rotational speed and subharmonics of critical frequencies). Theoretical fault frequencies depend on the rotational speed and structural effects such as material and axle model. If energy increases are detected at these frequencies, it is known to be due to the occurrence of the crack and not to other causes.
- Hypothesis 3: the energy increases that occur at the theoretical defect frequencies are significant enough to be detected by these signals and these signal processing techniques, therefore allowing the presence of a crack to be diagnosed.
- Hypothesis 4: these energy surges do not manifest themselves when the axle is not damaged.

**Figure 1:**
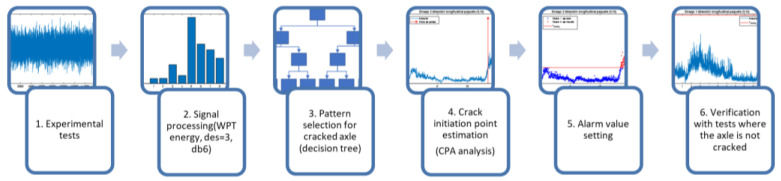
Flowchart of the proposed methodology.

To validate these hypotheses, an original methodology is proposed in Figure 1, comprising the following main steps:

1. Design and performance of the experimental tests. Experimental tests are designed in such a way that the natural frequencies of the specimen to be tested are obtained in the first place. On the other hand, some fatigue tests are designed and carried out on a railway axle fatigue testing machine. A triaxial accelerometer to register vibration signals is set ($f_{s}=6000\;\mathrm{Hz}$), and two classes of test are conducted: one where the railway axles fatigue limit is exceeded and there is evidence that they have been cracked during the test (magnetic particle testing), as well as axles in which the fatigue limit is not exceeded and there is evidence that they have not been cracked during the test. The crack under study is transverse and subsurface. This is a fatigue crack and appears below a section change, which is a stress concentration zone. These cracks are the most common cracks within railway axles, and this is the area where fatigue cracks would appear in the railway axle fatigue test rig used, which operates to perform railway axle fatigue tests according to the standard EN 13261:2009+A1:2010.
2. Processing of the vibration signals obtained by calculating the WPT energy, using the Daubechies 6 function as the mother wavelet and with initial decomposition level k = 3 (then, the signal is divided in $2^{k}=8$ packets of the same resolution, that is, $\Delta f=\frac{f_{s}}{2^{k+1}}=375\;\mathrm{Hz}$). This makes it possible to increase the manageability of the signals while retaining valuable information about the defect. This mathematical tool was chosen because of the good results it has provided in previous work on the detection of cracks in axles. With respect to the mother wavelet, in previous work carried out in this field, the Daubechies 6 mother wavelet has traditionally been used due to its good results. However, recent studies have shown that the mother wavelet function chosen does not greatly affect the results obtained [52]. However, although recent studies have shown that the mother wavelet function chosen does not greatly affect the results obtained, according to [53], using the Daubechies 6 mother wavelet improves reliability when detecting incipient defects.
3. Pattern selection. When there is evidence that the axle is cracked during the test, an analysis of the evolution of the energy with the number of cycles by frequency bands or packages is carried out. The aim is to find the packages whose energy value is sensitive to the appearance of the defect. Those packages that show a change of trend or change point in the energy levels that allow the crack initiation to be detected are chosen as possible defect indicators.

It is intended that the selected patterns or packets cover a band of frequencies narrow enough to identify the frequency to which it is related and also that it is not affected by events other than the defect. For this purpose, a decomposition level k = 9 ($2^{k}=512$ packets, $\Delta f=\frac{f_{s}}{2^{k+1}}=5.86\;\mathrm{Hz}$) was chosen. To automate the selection of patterns among the 512 possible, a decision tree was established, starting at decomposition level k = 3, where packets exceeding a threshold value in the final test area were chosen to be decomposed up to decomposition level k = 6 ($2^{k}=64$, $\Delta f=\frac{f_{s}}{2^{k+1}}=46.87\;\mathrm{Hz}$). Again, in this phase, those packets that exceed the threshold value established as a decision criterion at level 6 will be decomposed up to level 9.

The decision criterion within the tree to select the patterns will be the overcoming of a certain threshold value, defined in Equation (2).

(2) $$x_{threshold}=ref+3\sigma$$

Here, *ref* is the value that is set as the normal operating value of the machine and the value selected as the 98% value in the empirical cumulative probability density function, also called the Kaplan–Meier estimator. This conservative value was chosen for the threshold because it aimed to detect truly significant changes within the trend. The parameter *σ* represents the standard deviation of these values. A time window of *n* points corresponding to the start of the test, where it is considered that the crack has not appeared—i.e., representative of the steady-state operation of the machine before the appearance of the defect—is taken for the calculation of *ref* and *σ*. This criterion is based on the observed machine data and considers that when the energy levels are stable, the acceptable deviation is lower than if the fluctuation is high. This criterion for the decision tree was established according to the recommendations given in [54] for the establishment of automatic adaptive alarms.

4. Study of the crack initiation or change point. Once the packets indicating the presence of the crack have been selected, the offline change point will be determined using the clustering algorithm based on k-means ++. Euclidean distances are used after checking that the method of distance measurement does not affect the results. In this way, the data are divided into two groups (cracked axle–non-cracked axle), and the transition determines the change point, which defines the point where the crack is detectable using these techniques. This would confirm Hypothesis 3. The change point may vary from one packet to another depending on the sensitivity of the packets to the occurrence of the crack. The determination of the change point will serve to indicate which of the packets affected by the crack has a higher sensitivity to the occurrence of the crack and can therefore provide earlier detection. The pattern that shows the changeover point earliest will be considered the optimum pattern. The change point estimated in this way will establish the transition energy value $x_{alarm_{f}}$ to be considered as the alarm value, which will be validated through statistical techniques.
5. Parameterization of the automatic adaptive alarm value. Once the final alarm value $x_{alarm_{f}}$ has been obtained in each case, a parameterisation of the same is studied to stablish an alarm value appropriate to the response of the machine that can be calculated during an online test. The parameterization is carried out according to the sample mean and standard deviation of the energies for a time window of size *n*, previous to the point analysed. This is in accordance with the recommendations given in [54] for automatic adaptive alarms, and it is also in line with the establishment of the threshold value for pattern selection in decision trees (step 3 of methodology). The parametric adjustment of the weight of these two parameters is performed by calculating the minimum norm least squares solution to the indeterminate equation that yields the value of $x_{alarm_{f}}$ as a function of the mean and standard deviation.
6. Verification that the selected fault indicators do not exceed the set alarm value for tests where the axle is not cracked.

## 4. Experimental Setup

**Figure 2:**
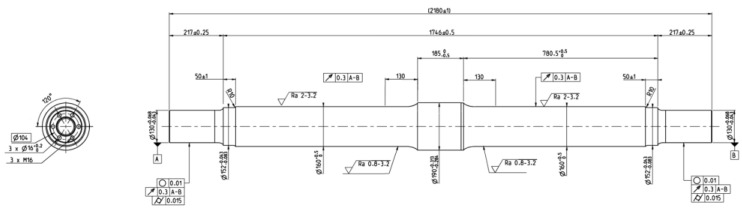
Details of dimensions of specimens used for fatigue testing.

The specimens tested were two EA4T (25CrMo4) hollow steel railway axles with the same geometry. Figure 2 details the dimensions of the specimens used for the tests.

**Figure 3:**
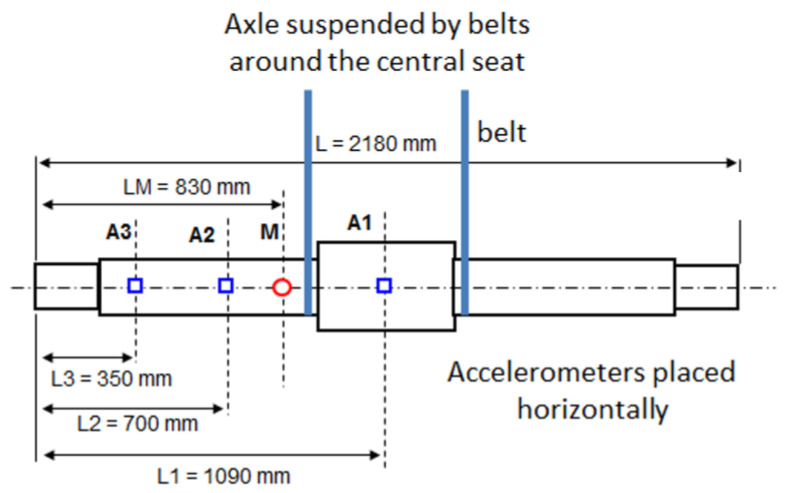
Experimental system for experimental modal analysis on the shaft used for fatigue testing. The accelerometers are located in sections A1, A2 and A3, and the dynamometric hammer impacts section M.

Before fatigue tests were carried out, an experimental modal analysis was performed to determine the natural frequencies. Three accelerometers and a dynamometric hammer were used for this purpose. Figure 3 shows the configuration used for the modal analysis.

The first five experimentally obtained natural frequencies are shown in Table 1.

**Figure 4:**
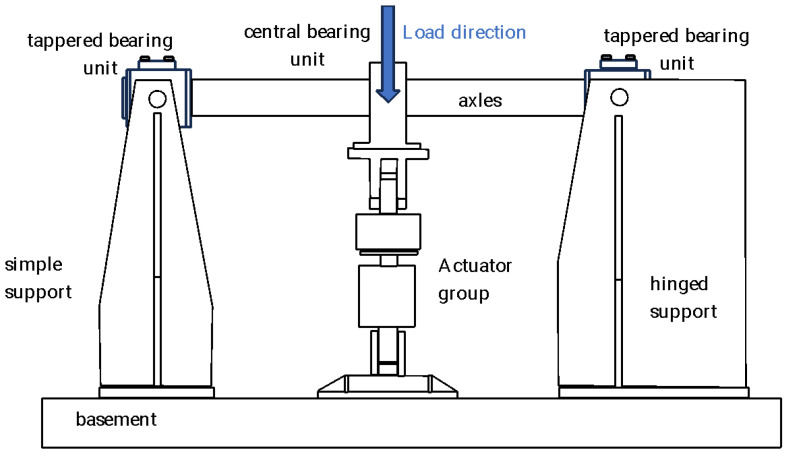
Diagram of the test rig used for the fatigue tests (adapted from [55]).

The test rig used for the fatigue tests was owned by Lucchini R.S. and located in a building owned by the Politecnico di Milano (Italy) at the time of the tests. The test rig was a horizontal machine where the shaft rotated on two tapered bearings and the loads were applied by using a pneumatic cylinder directly on the central bearing support. Figure 4 shows the scheme of the test rig.

Typically, before performing railway axle fatigue tests, maximum permissible stresses are calculated using fatigue limits and a safety parameter, which depend on the steel quality and on the application. Fatigue limits and their verification are established in manufacturing and qualification standard EN 13261:2009+A1:2010. Fatigue limits must be verified for axles, assuring that the axle is not cracked after 10^7^ cycles.

Two stopping criteria are used for fatigue tests: firstly, that 10^7^ cycles are reached; secondly, that before this value is reached, crack initiation is detected. Cracks are detected through measurements of shaft centre displacement, temperature and variation in applied load.

Two fatigue tests were performed with the load and speed characteristics given in Table 2. Test 2 was performed with a load above the fatigue limit, and the shaft cracked. In test 1, the load did not exceed the fatigue limit and therefore, the test was terminated at 10^7^ cycles.

The condition of the shafts was inspected after tests 1 and 2. Both ultrasonic and magnetic particle inspections were carried out, and at the end of test 1, the shaft was found to be free of defects. However, non-destructive inspection tests confirmed the presence of a crack in the shaft at the end of test 2.

**Figure 5:**
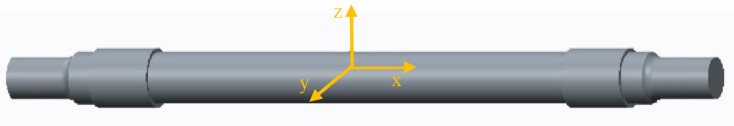
Accelerometer measurement directions: axial (*x*), longitudinal (*y*) and vertical (*z*).

Figure 5 shows the measurement directions of the accelerometer on the axle. As can be seen, an axial *x* direction and two radial directions were measured: the vertical *z* and the longitudinal *y* (parallel to the theoretical forward or track direction).

Prior to the start of the fatigue tests, an MMF triaxial accelerometer, model KS943B10, was installed in the central bearing housing. The accelerometer was connected to an MMF model M32 signal conditioner and a Keithley 3100 acquisition card. The parameters of the acquired signals are shown in Table 3.

Regarding the temporal resolution, a time waveform was taken every 3 min. The number of measured signals M for each test is shown in Table 4.

## 5. Results

Following the proposed method detailed in Figure 1, this section shows the results of the processing of the experimental data (step 1), starting from the cracked axle. The analysis (steps 2–4) of the faulted case allows us to determine the alarm threshold (step 5) and the subsequent validation against the healthy axle (step 6).

### 5.1. Experimental Tests

The experimental setup and the description of the tests have already been detailed in Section 4. Table 5 summarizes the main characteristic frequencies and principal harmonics that can be excited in the presence of a rotor crack.

### 5.2. Signal Processing

With reference to the cracked axle (test 2), the vibration signal was decomposed into eight packets by means of WP transform, and then the evolution of the energy within each packet was studied. Those packets that reached the initial alarm value were selected as patterns. In this study, the axial and vertical directions were discarded initially, and only patterns in the longitudinal direction were found.

**Figure 6:**
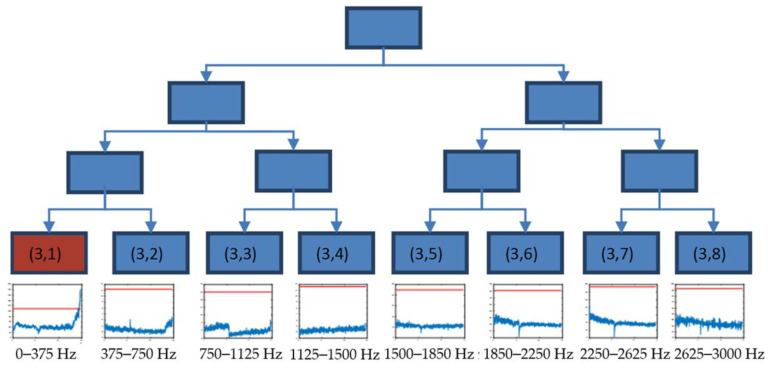
Decision tree until decomposition level 3, using the time evolution of energies (V^2^/Hz) and threshold value (red) $x_{threshold}$ in longitudinal direction for test 2.

Figure 6 shows the time evolution of the energy of longitudinal vibration for the eight packets obtained at decomposition level 3 with the initial alarm value stablished for each case (in red).

The energy of packet (3,1) (0–375 Hz) experienced a relevant change in the trend at the final part of test 2, exceeding the initial alarm value. Thus, this packet was selected in the decision tree to continue with a new decomposition.

### 5.3. Pattern Selection for Cracked Axle

**Figure 7:**
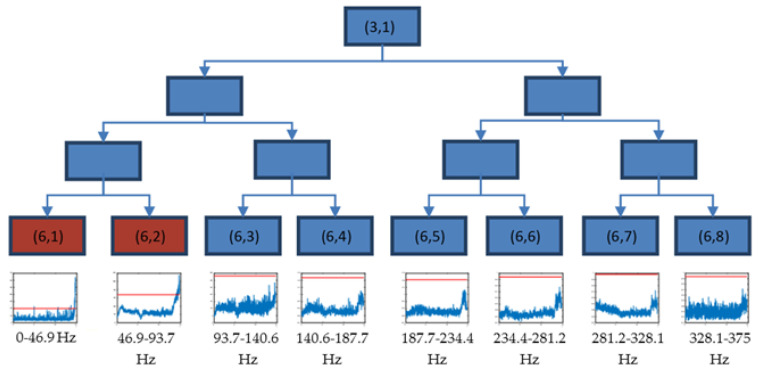
Decision tree for the decomposition of the packet (3,1) until level 6, using the time evolution of energies (V^2^/Hz) and threshold value (red) $x_{threshold}$ in the longitudinal direction for test 2.

Starting from the result of the previous step, packet (3,1) was decomposed until level 6 in eight packets with the same resolution. The decision tree of this decomposition is shown in Figure 7, where packets (6,1) and (6,2) are selected.

This way, packets (6,1) and (6,2) were selected to perform a new decomposition, each one in three levels. Thus, the frequency band 0–93.75 Hz was decomposed in 16 packets, each of the same resolution (Δf=5.9 Hz).

**Figure 8:**
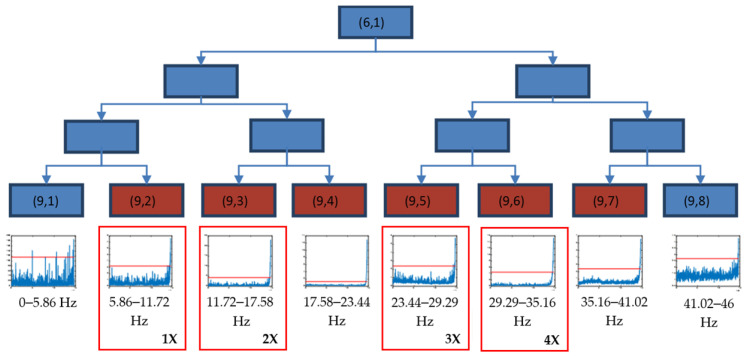
Decision tree for the decomposition of the packet (6,1) until level 9, using the time evolution of energies (V^2^/Hz) and threshold value (red) $x_{threshold}$ in the longitudinal direction for test 2.

Figure 8 shows the level 3 decomposition of packet (6,1), where the frequency band 5.86–41.02 Hz (packets from (9,2) to (9,7)) exceeded the initial alarm value during the final part of test 2, providing the reason for why these packets were selected as crack indicators. These frequencies were related to the rotating speed, (8.5 Hz), with the harmonics being 1×, 2×, 3× and 4× and their closest neighbours, due to reflections documented in [50].

**Figure 9:**
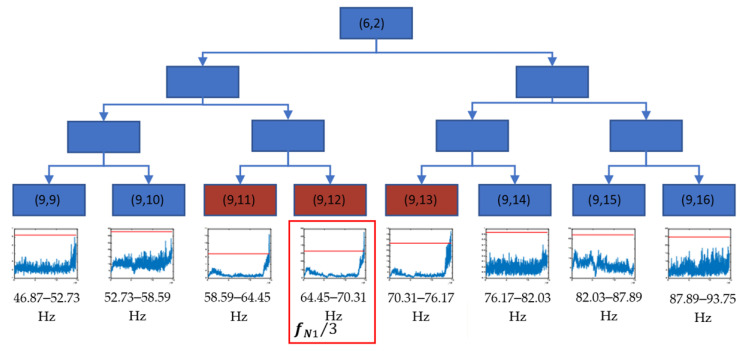
Decision tree for the decomposition of the packet (6,2) until level 9, using the time evolution of energies (V^2^/Hz) and threshold value (red) $x_{threshold}$ in the longitudinal direction for test 2.

Figure 9 shows the level 3 decomposition of packet (6,2). The frequency band related to 58.59–76.17 Hz (packets from (9,11) to (9,13)) showed an anomalous behaviour that started at the end of test 2. These packets were also clear crack indicators, being related to the first experimental natural frequency f_N1_ (1/3 f_N1_ and closest neighbours).

Considering these results, it can be affirmed that Hypotheses 1 and 2 are validated, since the appearance of the crack caused changes in certain energies in the vibratory signal that were experimentally detectable and could be associated with theoretical frequencies of defect appearance.

### 5.4. Crack Initiation Point Estimation

**Figure 10:**
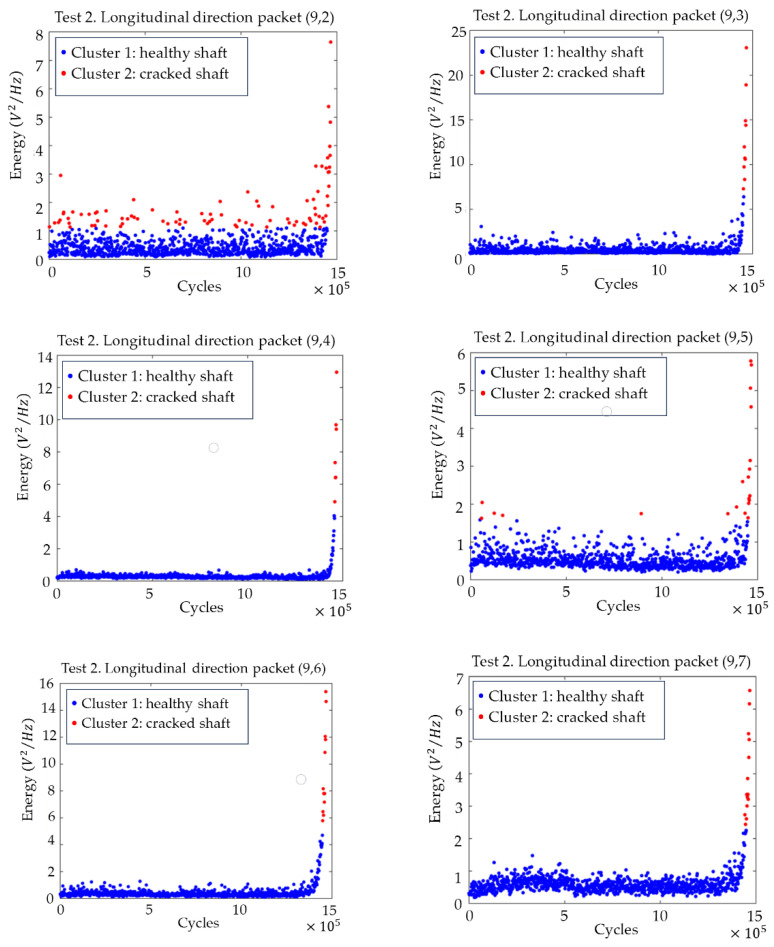
Time evolution of the energies for the packets from (9,2) to (9,7) related to the harmonics of the rotational speed, with the division into two clusters (cracked–non-cracked), in the longitudinal direction for test 2.

To find the time point where the crack started, a change point analysis was performed, applying clustering based on the “k-means++” algorithm. To find the change point, two clusters were used, the change point being the transition between them. Figure 10 shows the graphs with the evolution of the energies and the change point calculated with this algorithm for the packages (9,2) to (9,7). The clearest transition appears in packages (9,3), (9,4), (9,6) and (9,7), where the changeover point is clear for all four cases approximately 15,000 cycles before the end of the test, which implies a test time of 30 min.

**Figure 11:**
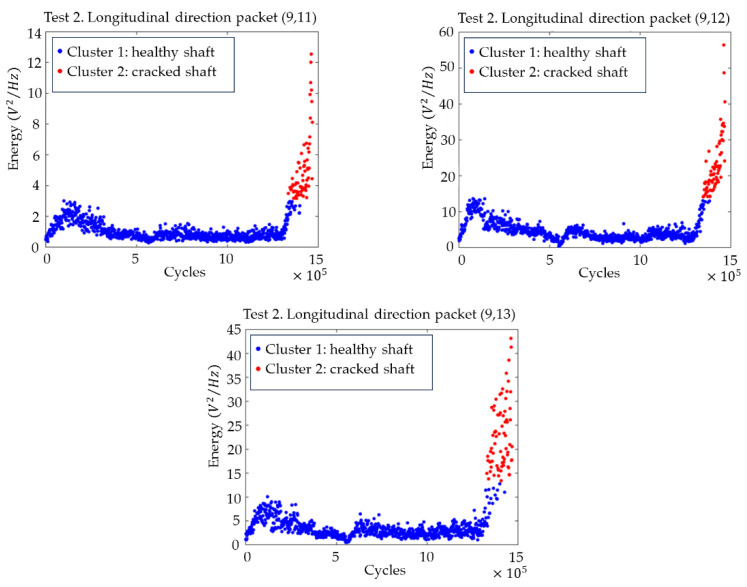
Time evolution of the energies for the packets from (9,11) to (9,13) related to the subharmonics of the natural frequencies of the shaft, with the division into two clusters (cracked–uncracked), in the longitudinal direction for test 2.

Figure 11 shows the plots with the evolution of the energies of the (9,11), (9,12) and (9,13) packages, as well as the changeover point calculated using clustering. For all three packages, the transition is clear, and the changeover point can be located approximately 115,000 cycles before the end of the test, which implies almost 4 h in time.

**Figure 12:**
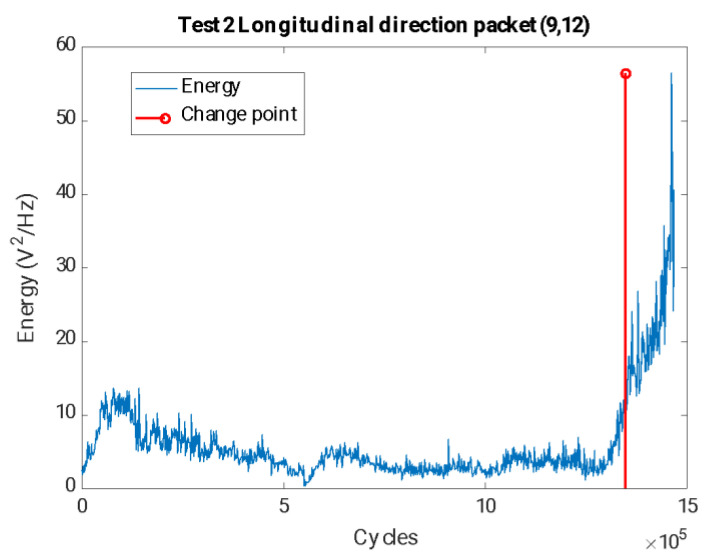
Time evolution of the energies for the packet (9,12) related to the 1/3 subharmonic of the first natural frequency of the shaft, together with the crack initiation estimated by splitting into two clusters (cracked–uncracked), in the longitudinal direction for test 2.

In view of the results, it can be concluded that the frequency related to the 1/3 subharmonic of the first natural frequency of the axle fN1 is the one that is most sensitive to the appearance of the crack, showing greater changes in its energy levels and occurring earlier. Based on the grouping for the packages related to these frequencies, it can be affirmed that the crack can be seen after 1,352,172 cycles of the machine, which, at the speed of the test, corresponds to about 44 h, as can be seen in Figure 12 with the package (9,12) (related to the band 64.41–70.31 Hz).

The selection of the change point also sets the threshold value set $x_{threshold_{i}}$ to determine that a crack exists, which, in the case of the (9,12) package, takes an energy value of 14.16 V^2^/Hz.

**Figure 13:**
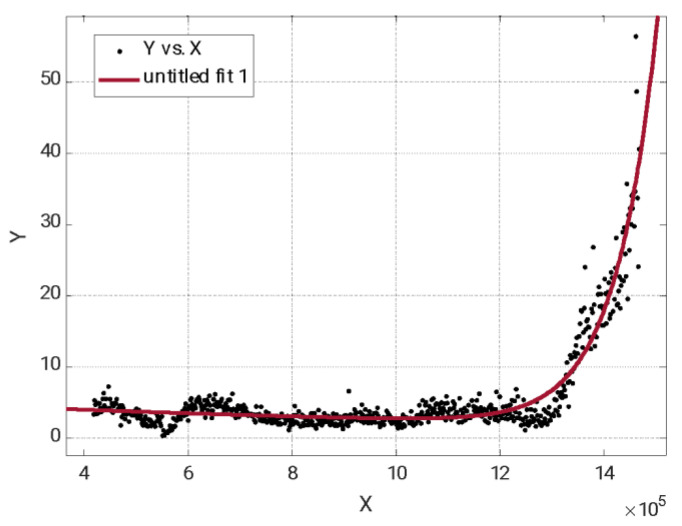
Evolution of the energy (V^2^/Hz) with the number of cycles for the packet (9,12), with the experimental data against an exponential curve. R-square = 0.9.

To validate the selected alarm value, the probability that the data corresponding to a safe area will exceed the threshold value, corresponding to data where the crack has not started, was studied. To perform this calculation, it was assumed that the population of data with respect to the number of cycles was adjusted with an R-square of 0.9 to an exponential function, according to Figure 13.

This allows us to assume that the value of the package energy (9,12) versus the defect size has a lognormal correlation and that two zones are clearly differentiated: the first one where the crack is not present or not detectable and a second zone where the crack is detectable and the energy of the packet grows exponentially.

Assuming this distribution, if data corresponding to a safe zone where the crack has not appeared are taken, the probability of obtaining a false alarm is less than 0.01%.

Therefore, Hypothesis 3 can be considered validated, since the changes detected in the energies are of sufficient magnitude to establish an alarm value for the detection of the crack.

### 5.5. Alarm Value Setting

The clustering offers different energy values as a threshold value for the healthy axle–cracked axle transition for each analysed packet *i* ($x_{threshold_{i}}$). However, the aim was to predict a threshold value as an alarm for an online process from the values given by the machine in each test. With the data obtained for the change point or crack initiation from the “k-means++” clustering algorithm for packets (9,11), (9,12) and (9,13), a parametric adjustment of the alarm value was performed to make it a function of the cumulative mean for packet *i* ($\mu_{i}$) and the cumulative standard deviation for package *i* ($\sigma_{i}$). The values $\mu_{i}$ and $\sigma_{i}$ were calculated for the energies obtained from the end of the running-in period, which was around 400,000 cycles and up to 1,200,000 cycles in view of the data, at which point the crack had not manifested itself.

The fit for each case was performed by calculating the minimum norm least squares solution for a and b in Equation (3).

(3) $$\left[\mu_{i}\;\sigma_{i}\;\right]\left[a_{i}\;b_{i}\;\right]=x_{threshold_{i}}$$

For packets (9,11), (9,12) and (9,13), the solutions for $a_{i}$ and $b_{i}$ are given in Table 6.

For the different packets analysed, the $a_{i}$ and $b_{i}$ values were similar. To improve the data separation of the two groups and thus minimize the number of points needed to confirm the change point in an online method, the parameters a and b were set as the maximum obtained. Thus, the final fitted alarm was calculated according to Equation (4).

(4) $$x_{alarm_{f}}=3.7\mu +1.25\sigma$$

**Figure 14:**
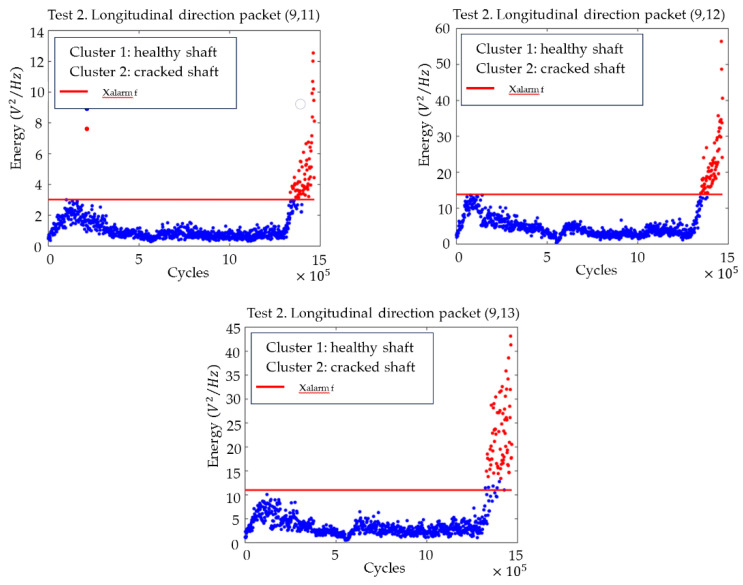
Time evolution of the energies for the packages from (9,11) to (9,13) related to the subharmonics of the natural frequencies of the axis, with the division into two clusters (cracked–uncracked) and with the adjusted final alarm value $x_{alarm_{f}}$ in the longitudinal direction for test 2.

Figure 14 shows the time evolution of the energies for the packets selected as the best flaw indicators, together with the final set alarm value that would serve to establish a crack detection criterion or a stop criterion for the test.

Parametric adjustment of the final alarm value $x_{alarm_{f}}$ has shown that it is possible to use this value as a threshold to detect the crack initiation in an online process.

### 5.6. Validation against Uncracked Axle

**Figure 15:**
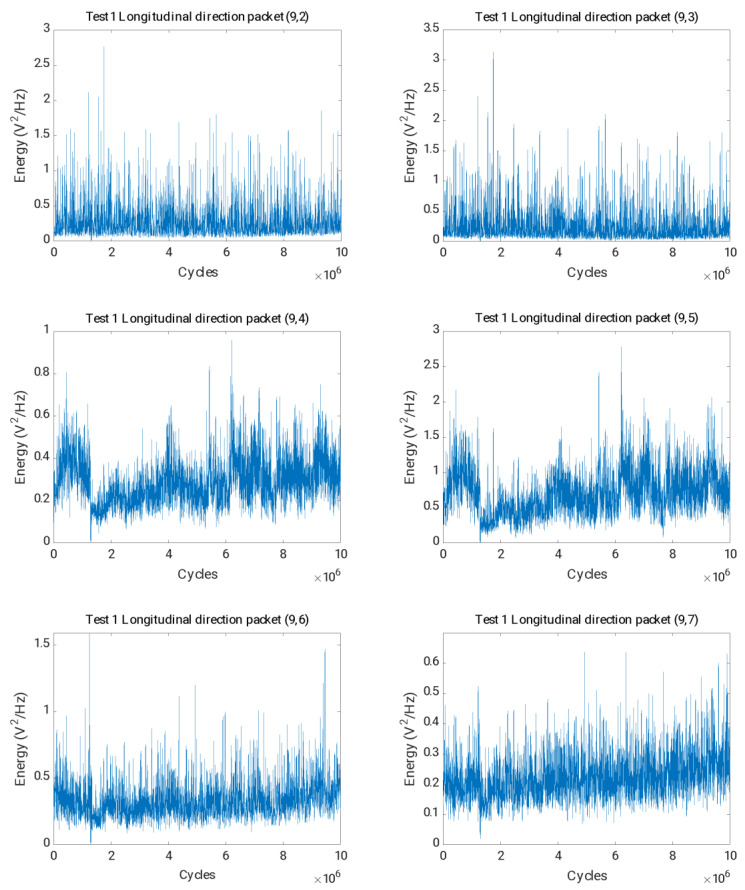
Time evolution of the energies for the packets (9,2) to (9,7) in the longitudinal direction for test 1.

In the final step, the parameters chosen as crack indicators for test 2 were used to analyse data from test 1, in which the axle was not cracked. Figure 15 shows the evolution of the packet energy related to the harmonics of the rotational speed, from (9,2) to (9,7). It can be concluded that the trend of the energies was stationary in mean and variance throughout the test, and the effects observed in the case where the crack appeared were not observed.

**Figure 16:**
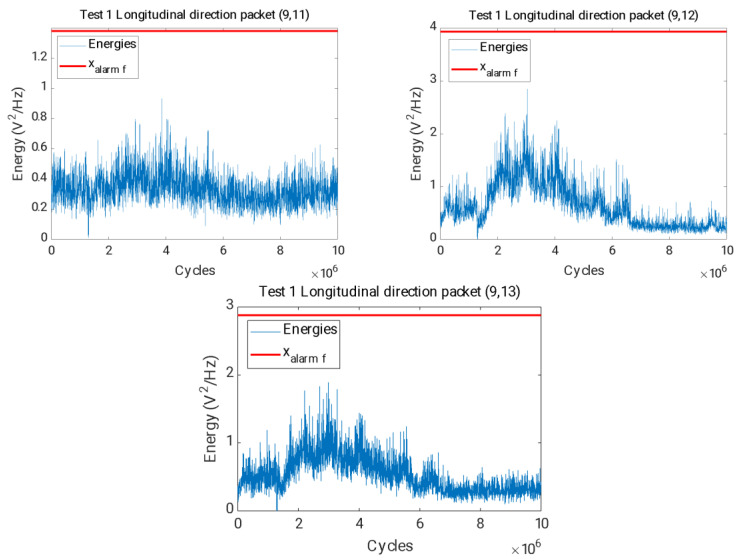
Time evolution of the energies for the packages (9,11), (9,12) and (9,13) and final alarm value $x_{alarm_{f}}$ in red in the longitudinal direction for the test.

Figure 16 shows the evolution of the energy of the packets related to the 1/3 subharmonic of the first natural frequency of the axis, from (9,11) to (9,13), where it can be observed that their energies have a stable trend and do not exceed the alarm value established for these packets.

This allows Hypothesis 4 to be confirmed, as the changes observed during test 2, where the shaft was cracked, are not apparent during test 1, where the shaft did not break.

## 6. Conclusions

This paper presents a methodology for automatic crack detection in railway axles. First, experimental vibration signals were taken during fatigue tests of two identical specimens. In one of them, the test load exceeded the fatigue limit and it therefore cracked; in the other one, the fatigue limit was not exceeded and the test ended with a healthy axle.

For the test where the axle was cracked, the evolution of the WPT energy related to selected frequencies was analysed by working on a decision tree. In this study, trend changes were observed in the energies of the final part of the test at the theoretical defect frequencies related to the harmonics of the rotational speed and subharmonics of the shaft eigenfrequencies, confirming Hypothesis 1 and 2. The most sensitive indicator of crack initiation turned out to be the energy of the frequency related to the 1/3 subharmonic of the first natural frequency of the axle, obtained experimentally. These WPT energy increases are sufficient for crack detection using automatic adaptive alarm values based on the “k-means ++” clustering, confirming Hypothesis 3. This phenomenon does not occur when the axle is not damaged, confirming Hypothesis 4.

The use of the WPT approach due to its filtering capability facilitates the study of patterns and its association with theoretical fault frequencies. Furthermore, it allows us to be sure that energy increases are due to cracking and not to other effects. It is also very useful for the study of the optimal patterns for crack detection in the proposed methodology, promoting an early diagnosis. On the other hand, the establishment of an automatic adaptive alarm value allows generalisation, as it is based on the mean and standard deviation of the machine’s data, allowing it to adapt to different models and operating conditions, as well as dealing with the problem of condition monitoring in non-stationary conditions. This would solve a problem already reported in previous work where supervised learning classification systems were used. The validation of the methodology was performed on the basis of vibration signals obtained during fatigue tests of real full-scale railway axles where fatigue cracks were naturally occurring, which is also valuable. In addition, various vibration directions were measured, and we concluded that indications of cracking occurrence were mainly in the longitudinal direction of the vibration (parallel to the track).

This approach gives promising results to complement current inspection techniques, since specific information related to the crack can be monitored between inspection intervals, improving safety.

As for future work, the proposed approach could be extended to consider other positions for the crack and other types of faults, such as bearing faults or wheel defects. Also, new segmentation possibilities or new indexes in clustering could be studied. Related to artificial intelligence, deeper studies can also be conducted to try to solve the lack of generalisation reported in previous works—using Support Vector Machines or Artificial Neural Networks, for example. Finally, field tests could be performed to validate the performance of the proposed method in real-world scenarios.

## Figures and Tables

**Table 1:** First five natural frequencies obtained from experimental modal analysis.

Natural Frequencies N	Frequency [Hz]
f_N1_	194.2
f_N2_	485.2
f_N3_	886.0
f_N4_	1335.5
f_N5_	1857.2

**Table 2:** Characteristics of fatigue tests performed, including load and speed, as well as stopping reason and number of cycles.

Test	Speed (r.p.m.)	Load (kN)	Stopping Reason	Number of Cycles
1	509	216	Number of cycles	10,000,000
2	509	240	Alarm values reached	1,467,172

**Table 3:** Parameters of acquired signals.

Sampling Frequency (Hz)	Signal Length (N)
6000	16,384 (2^14^)

**Table 4:** Number of acquired signals (M) for each test.

Test	Speed (r.p.m.)	Number of Cycles	Number of Signals (M)
1	509	10,000,000	6550
2	509	1,467,172	960

**Table 5:** Main characteristic frequencies and principal (sub)harmonics.

Rotating Frequency (Hz)	2X	Harmonics (Hz) 3X	4X
8.48	16.97	25.45	33.93
	**1/2X**	**Subharmonics (Hz)** **1/3X**	**1/4X**
194.2	97.1	64.73	48.55

**Table 6:** Parametric setting of $a_{i}$ and $b_{i}$ for the establishment of an online alarm value.

Packet *i*	$a_{i}$	$b_{i}$
(9,11)	3.7	1.12
(9,12)	3.65	1.25
(9,13)	3.33	1.2

## Data Availability

The data presented in this study are available on request from the corresponding author.
